# Spatial Profiling of the Prostate Cancer Tumor Microenvironment Reveals Multiple Differences in Gene Expression and Correlation with Recurrence Risk

**DOI:** 10.3390/cancers14194923

**Published:** 2022-10-08

**Authors:** Vinay Kumar, Pavneet Randhawa, Robert Bilodeau, Dan Mercola, Michael McClelland, Anshu Agrawal, James Nguyen, Patricia Castro, Michael M. Ittmann, Farah Rahmatpanah

**Affiliations:** 1 Department of Pathology and Laboratory Medicine, University of California, Irvine, CA 92697, USA; 2 Department of Molecular and Microbiology, University of California, Irvine, CA 92697, USA; 3 Department of Medicine, University of California, Irvine, CA 92697, USA; 4 Department of Pathology & Immunology, Baylor College of Medicine, Houston, TX 77030, USA

**Keywords:** prostate cancer, tumor microenvironment, Digital Spatial profiling, OX40L, CTLA4

## Abstract

**Simple Summary:**

In prostate cancer (PCa), the tumor microenvironment plays a crucial role in both the development and progression of the disease. We used Digital Spatial Profiling multiplex technology to assess the expression of 58 protein and 1825 RNA transcripts in traditionally challenging samples (i.e., archived FFPE samples) while maintaining the spatial context of expression. First, we identified differences in protein and RNA expression among multiple cell types (stromal, epithelial, and immune cells). Second, we used a PCa tissue microarray of 1547 cores (97 patients) to further validate the protein expression of selected genes in a larger cohort of prostate cancer patients. We also computed survival models testing the relationship between OX40L, CTLA4, and CD11c protein expression in both tumor and tumor-adjacent stroma samples with time to biochemical relapse.

**Abstract:**

The tumor microenvironment plays a crucial role in both the development and progression of prostate cancer. Furthermore, identifying protein and gene expression differences between different regions is valuable for treatment development. We applied Digital Spatial Profiling multiplex analysis to formalin-fixed paraffin embedded prostatectomy tissue blocks to investigate protein and transcriptome differences between tumor, tumor-adjacent stroma (TAS), CD45+ tumor, and CD45+ TAS tissue. Differential expression of an immunology/oncology protein panel (*n* = 58) was measured. OX40L and CTLA4 were expressed at higher levels while 22 other proteins, including CD11c, were expressed at lower levels (FDR < 0.2 and *p*-value < 0.05) in TAS as compared to tumor epithelia. A tissue microarray analysis of 97 patients with 1547 cores found positive correlations between high expression of CD11c and increased time to recurrence in tumor and TAS, and inverse relationships for CTLA4 and OX40L, where higher expression in tumor correlated with lower time to recurrence, but higher time to recurrence in TAS. Spatial transcriptomic analysis using a Cancer Transcriptome Atlas panel (*n* = 1825 genes) identified 162 genes downregulated and 69 upregulated in TAS versus tumor, 26 downregulated and 6 upregulated in CD45+ TAS versus CD45+ tumor. We utilized CIBERSORTx to estimate the relative immune cell fractions using CD45+ gene expression and found higher average fractions for memory B, naïve B, and T cells in TAS. In summary, the combination of protein expression differences, immune cell fractions, and correlations of protein expression with time to recurrence suggest that closely examining the tumor microenvironment provides valuable data that can improve prognostication and treatment techniques.

## 1. Introduction

Prostate cancer is the fifth leading cause of death among males worldwide, with a higher prevalence in developed countries [1]. Various drugs have been approved by the FDA to target tumor cells; however, many have not been successful in treating cancer due to their inability to address the role of tumor microenvironment components [2]. Multiple studies support the idea that the tumor microenvironment—including tumor cells, stromal tissue, immune cells, and the extracellular matrix—plays an important role in the development and progression of prostate cancer [3,4,5,6].

Prostate cancer arises from the epithelial component of the prostate gland, but studies have shown that tumor-adjacent stroma also plays a role in tumorigenesis [6]. Our prior studies have indicated that a prostate tumor-adjacent stroma has hundreds of significant RNA expression differences from normal stroma tissue [7]. We used these properties to develop biomarker panels that reliably distinguish a normal prostate from tumor-bearing prostates or distinguish good and bad disease outcomes for individual patients [8,9,10]. These observations illustrate the potential of stroma characterization in assisting with the management of prostate cancer [11,12,13,14]. Moreover, the type, location, and density of immune cells within the tumor site has been reported to be a better predictor for survival than classical tumor staging [15].

Currently, protein expression in formalin-fixed, paraffin embedded (FFPE) tissue is measured using immunohistochemistry analysis, but despite its popularity, the technique is not precise and offers limited information about specific targeted regions [16]. Moreover, obtaining high quality data from archived FFPE samples is challenging. The GeoMx Digital Spatial Profiling (GeoMx DSP, NanoString) technology allows a multiplex assessment of protein and RNA expression in challenging samples while maintaining the spatial context of the expression [17]. We applied DSP to FFPE tissues from four prostate cancer patients to identify region-specific differences within tumor-adjacent stroma, tumor, and CD45+ tumor-adjacent stroma and CD45+ tumor cells. CD45 is a marker for all types of immune cells and can detect immune cell infiltration into tumor and stroma [18]. We used a PCa tissue microarray to further validate the protein expression of selected genes in a larger patient sample. We also computed survival models testing the relationship between the percent positivity of OX40L, CTLA4, and CD11c expression in both tumor and stroma samples with time to biochemical relapse.

## 2. Materials and Methods

### 2.1. Patient Characteristics and Sample Selection

Formalin fixed, paraffin embedded (FFPE) prostatectomy tissue blocks (*n* = 4) were obtained by informed consent using Institutional Review Board (IRB)-approved and HIPPA-compliant protocols at the Baylor College of Medicine (BCM) (Supplementary Table S1). All four samples were collected prior to the treatment of the patients and matched for clinical variables (i.e., biochemical relapse, age, Gleason score, and tumor stage).

### 2.2. Digital Spatial Profiling (DSP)

NanoString’s Digital Spatial Profiling (DSP) technology uses digital optical barcoding and immunofluorescence techniques to indicate tissue morphology and select specific regions of interest for multiplexed spatial profiling in both RNA and protein targets. Four prostate cancer tissue blocks from four prostate cancer patients were processed for spatial transcriptomic and proteomic analysis by NanoString Inc (Seattle, WA, USA). Briefly, the FFPE tissue sections were stained with four morphology markers (fluorescent antibodies): epithelial (pan cytokeratin, PanCK), stromal (alpha smooth muscle-actin, αSMA), immune cell markers (protein tyrosine phosphatase, receptor type C, CD45), and nuclear markers (DNA). The sections were then combined with oligo-tagged antibodies (*n* = 58) for the protein panel and oligo-tagged ISH probes (*n* = 1825) targeting immune and cancer genes for the transcriptome panel. All GeoMX DSP antibodies are validated for sensitivity and reproducibility according to manufacturer specifications and recent publications [19,20].

Next generation sequencing (NGS) was used to analyze the photocleaved oligonucleotide tags for RNA. One advantage of the NGS readout over the nCounter readout system is its ability to assess several independent measurements per RNA transcript target through the tiled probes [21]. Twelve regions of interest per FFPE sample were selected and represent three tumor epithelium, three tumor-adjacent stroma, three CD45+ tumor epithelium, and three CD45+ tumor-adjacent stroma samples across four prostate cancer patients. The protein panel and cancer transcriptome panel were analyzed for each ROI across patient sample. All data collection and comparative statistical analysis was performed using NanoString GEO’s Mx software, GraphPad, and in R.

### 2.3. Tissue Microarray (TMA) Preparation

Nine prostate cancer tissue microarrays consisting of 97 patients (*n* = 1547 cores) were obtained from the Pathology Department at the University of California, Irvine as previously reported [22].

Blocks from each TMA were stained with three antibodies—CD11c, OX40L, and CTLA4 by CrownBio (Per CrownBio Inc., San Diego, CA, USA). Immunohistochemistry was performed on a Bond RX autostainer (Leica Biosystems) with heat induced epitope retrieval in an EDTA buffer (pH 9.0) using the standard protocol. The primary antibodies used were rabbit monoclonal CD11c antibody (Abcam, ab52632, 1:100), rabbit monoclonal OX40L antibody (Cell Signaling Technology, 59036, 1:100, Danvers, MA, USA), and rabbit monoclonal CTLA4 antibody (Abcam, ab237712, 1:100, Cambridge, UK). Bond polymer refine detection (Leica Biosystems, DS9800, Wetzlar, Germany) was used as a secondary antibody detection system according to the manufacturer’s standard protocol. After staining, sections were dehydrated and film coverslipped using a TissueTek-Prisma and Coverslipper (Sakura). Whole slide scanning (40×) was performed on a NanoZoomer Digital Slide System NDP2.0-HT (Hamamatsu, Shizuoka, Japan).

### 2.4. Tissue Microarray (TMA) Analysis

To reduce variation in expression analysis, cores were processed by a trained model for QuPath [23] (Supplementary File S1) to quantify cells as tumor-adjacent stroma and tumor epithelium. Cell positivity was calculated by adjusting intensity quantification to assign positivity for a calibration core. As multiple cores were linked to a single patient, cell counts were quantified and summed to generate a total count for each patient. The stained percentages were calculated by dividing the number of positive cell detections by the total number of cell detections in tumor and tumor-adjacent stroma, respectively, as we described previously [24].

Using the previously consolidated patient samples, percent positivity was normalized using a z-score transform as previously described [24]. Cutoff values for high and low expression thresholds were calculated using the *survminer* R package. The expression threshold evaluations were used to generate a Kaplan–Meier survival model. Two survival models focused on high and low positivity in tumor and tumor-adjacent stroma, respectively, were constructed for all three staining (OX40L, CD11c, and CTLA4), resulting in a total of 6 survival models.

### 2.5. Statistical Analysis of Cancer Transcriptome Atlas Panel, in Situ Detection of mRNA with Digital Spatial Profiling

Raw probe counts from the Cancer Transcriptome Atlas (CTA) panel underwent sequencing quality control (QC) using gene expression counts from each region of interest where regions that were found to be under-sequenced were dropped from further analysis. Probe QC identified each mRNA that was targeted by multiple and any outlier probes were subsequently removed from downstream analysis. The remaining data underwent signal based Q3 normalization in NanoString GeoMx software [17,25], and individual counts were normalized against the 75th percentile of signal from their own areas of interest. Similar to the protein panel, four morphology markers were used to validate the regions of interest in the CTA panel: PanCK/KRT18, SMA/ACTA, and CD45. Of the 1825 genes selected for the initial analysis, 676 were selected based on a limit of quantification (LOQ) greater than 20% of the region of interest for each gene. The formula used to calculate the signal to LOQ ratio is as follows: LOQ = GeoMean(NegProbes) ∗ GeoSD(NegProbes)^2.5^ [17,21].

### 2.6. Cell-Type Identification by Estimating Relative Subsets of RNA Transcripts

(CIBERSORTx) [26] was used to characterize the immune cell type composition of each ROI based on the gene transcript levels. Averaged normalized expression values for each immune-infiltrated (CD45+ tumor-adjacent stroma, *n* = 12; CD45+ tumor epithelium, *n* = 12) ROI were provided as an input for CIBERSORTx and yielded immune fraction scores for the 22 immune cell types pre-designated for the program.

### 2.7. Pathway Analysis

Statistically significant differentially expressed genes for tumor-adjacent stroma versus tumor epithelium and CD45+ tumor-adjacent stroma versus CD45+ tumor epithelium were analyzed using the Ingenuity Pathway Analysis (IPA) software (QIAGEN Inc., Germantown, MD, USA). The “Core Expression Analysis” module was used as previously described [24,27,28,29,30] to interpret the canonical pathways corresponding to the uploaded gene list. Significantly upregulated and downregulated canonical pathways were ranked according to their −log_10_(*p*-value) > 1.3 or a *p*-value < 0.05.

## 3. Results

Prior studies involving tumor and tumor-adjacent stroma (TAS) tissue have shown that deregulation of genes in TAS compared to tumor epithelium contribute to the increased aggressiveness of prostate cancer by altering immune responses in the tumor microenvironment [3,6,7,10]. Differences in the gene expression profiles, genomic aberrations, and molecular features in the tumor microenvironment may allow for the creation of new therapeutic treatments targeting the microenvironment [31,32].

Digital Spatial Profiling (DSP) allows for the simultaneous spatial analysis of tissues content of both protein and RNA targets in regions of interest (ROIs) without the interference of FFPE degradation and guided by fluorescence staining of known tissue markers [17,25,33,34]. We applied the NanoString GeoMx DSP multiplex assay to multiple regions of the prostate in four prostate cancer (PCa) patients (Supplementary Table S1).

### 3.1. Digital Spatial Profiling (DSP) of 58 Proteins from an Immune/Oncology Panel in the PCa Tumor Microenvironment

Overall, 48 regions of interest (12 tumor-adjacent stroma and 12 tumor epithelium, 12 CD45+ tumor and 12 CD45+ tumor-adjacent stroma) were identified in 4 tumor samples from four different prostate cancer patients using florescence morphology markers of epithelial, stromal and immune cells (i.e., PanCK, αSMA, and CD45, respectively). The regions of interest (ROIs) were then combined with oligo-tagged antibodies (*n* = 58) for the protein panel and oligo-tagged ISH probes (*n* = 1825) targeting immune and cancer genes for the transcriptome panel.

Overall, 58 immunology/oncology related proteins were analyzed in the 48 regions of interest. We examined multiple techniques to normalize the protein expression data [17], including using the three housekeeping genes (GAPDH, Histone H3, and Ribosomal protein) as normalized controls, using the three IgG control isotypes (Ms IgG1, Ms IgG2a, and Rb IgG) as a basis for a Signal to Noise normalization, or using ROI area and nuclei count to calculate an area normalization. The Signal to Noise normalization using the three IgG control isotypes was found to be the most appropriate normalization method for the protein data because, together, they had a concordance greater than 96% (Supplementary Figure S1).

**Figure 1:**
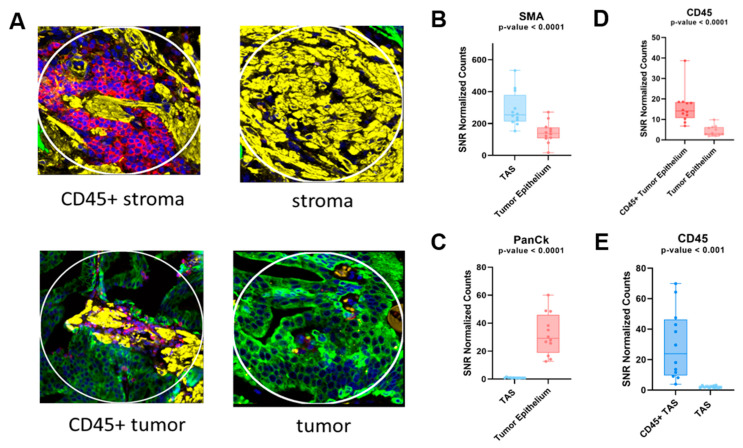
Immunofluorescence-stained cores for prostate cancer samples and scatter plots representing protein expression of tissue markers. DSP was used to quantify expression levels for each ROI. (**A**) Representative immunofluorescence-stained cores for each tissue type. Yellow staining represents the presence of αSMA, green represents PanCk, and red represents CD45. (**B**,**C**) Protein expression levels of αSMA and PanCK as an indicator of stromal tissue and tumor epithelial, respectively. (**D**,**E**) CD45 expression differences between CD45+ tumor epithelium and tumor epithelium as well as CD45+ TAS and TAS, respectively. Each dot represents an ROI. DSP = Digital spatial profiling; ROI = region of interest; TAS = tumor-adjacent stroma.

**Figure 2:**
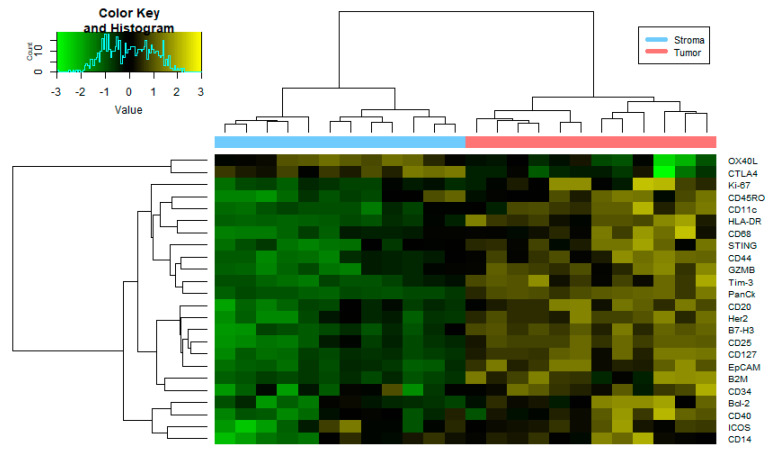
Heatmap of protein expression differences between stroma and tumor tissue. Heatmap generated using the normalized expression values for 24 proteins from an immunology/oncology panel (FDR < 0.2 and *p*-value < 0.05) that were differentially expressed among 12 tumor ROIs and 12 tumor-adjacent ROIs. See Supplementary Table S2 for numerical data. I/O = immune/oncology; FDR = false discover rate; ROI = region of interest.

Following normalization, 18 of the 58 immunology/oncology-related proteins analyzed were excluded from downstream analysis due to having a Signal to Noise Ratio (SNR) < 3.0 (Supplementary Figure S2). To determine whether the ROIs were confidently identified, we first analyzed the protein expression levels of PanCK, αSMA, and CD45 (Figure 1A). As expected, protein expression correlated with the observed morphology of each target region (Figure 1B–E). When tumor-adjacent stroma (12 ROI) was compared to tumor epithelium (12 ROI), 24 of 40 were found to be differentially detected at statistically significant levels between TAS and tumor epithelium at an FDR < 0.2 and a *p*-value < 0.05 across the four PCa patients (Figure 2, Supplementary Table S2). The 12 ROIs for each cell type clustered together across four PCa patients, verifying the reproducibility of ROIs across the same cell types.

In addition to the tumor epithelium marker PanCK, Granzyme B which is required for cytotoxic action of CD8+ T cells is increased at significantly higher levels in tumor when compared to tumor-adjacent stroma (TAS). It is now known that Granzyme B expression is not unique to natural killer and cytotoxic T cells; it can be expressed by both other immune cells and various other cell types [35,36].

In addition to CD11c, antigen presenting (HLADR and β2M), costimulatory molecules (CD40, ICOS) as well as STING and CD68 were all upregulated in tumor compared to TAS (Figure 2). The increased expression of HLADR, CD68, STING and β2M along with enhanced CD40 and ICOS is indicative of enhanced immune responses [37,38,39,40,41,42]. While the above-mentioned markers support immune activation, we also find upregulation of some inhibitory costimulatory molecules in the tumor as compared to TAS. These include the expression of other immune check point inhibitors such as TIM3 and B7-H3 [43,44].

As expected, the TAS identification marker αSMA was found to be overexpressed in TAS tissue when compared to tumor. Only two genes, OX40L and CTLA4, had significantly higher protein expression in TAS as compared to tumor (FDR < 0.2 and *p*-value < 0.05) (Figure 2, Supplementary Table S2). In breast cancer microarrays, high detection levels of OX40L in a subset of carcinoma associated fibroblast (main component of the stroma) has been associated with the retention of regulatory T cells (i.e., CD4 + CD25+ T cells) [45]. Furthermore, OX40L was also identified to be enriched at immune checkpoints in blastic prostate cancer during Digital Spatial Profiling analysis [46]. Protein and RNA expression of CTLA4 has also been reported on the surface of mesenchymal stem/stromal cells (non-immune cells) [47].

When comparing the expression of CD45+ TAS to CD45+ tumor, few proteins were found to be differentially detected among the samples. A total of four proteins were downregulated in CD45+ TAS compared to CD45+ tumor epithelium (Supplementary Table S3). These were PanCk, a marker for tumor epithelium, HER2, EPCAM, and B7-H3. B7-H3 has been identified to express at low levels in lymphoid cells and exhibits higher expression when induced, suggesting that it may play a role in tumor development [48].

### 3.2. Recurrence Risk Assessment of Selected Immune Markers in PCa Patients

**Figure 3:**
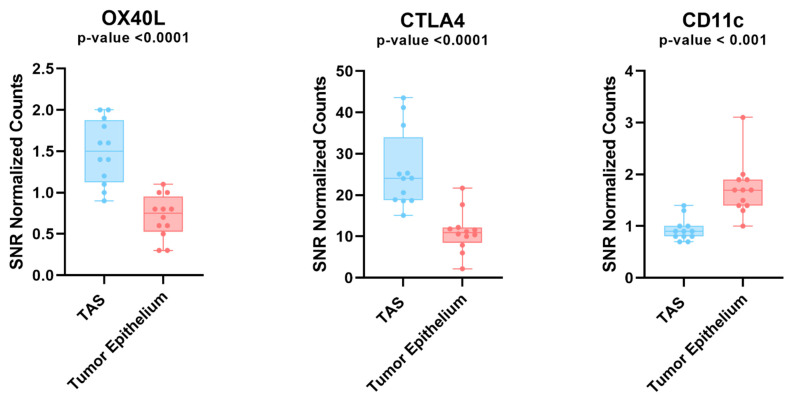
Protein expression analysis in tumor and TAS tissue across 4 prostate cancer patients. Box plot depicting the protein expression levels of OX40L, CTLA4, and CD11c. These genes all had significant differences between tumor and TAS. OX40L and CTLA4 were the only two proteins that were upregulated in tumor adjacent stroma compared to tumor. Each dot represents an ROI. TAS = tumor-adjacent stroma; ROI = region of interest.

**Figure 4:**
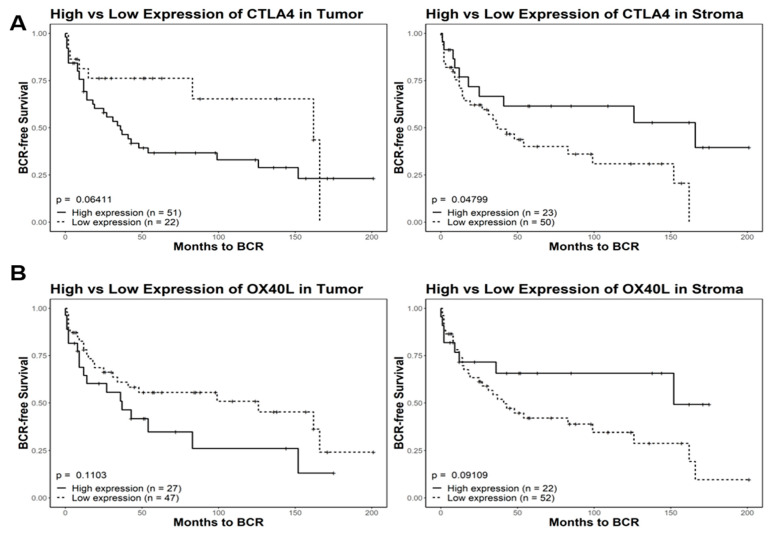
Kaplan–Meier survival analysis of CTLA4 and OX40L in tumor and tumor-adjacent stroma. Comparisons include high and low expression of CTLA4 (**A**) and OX40L (**B**). Quantized percent positivity from a series of TMA cores (n = 1547) was calculated using QuPath. Using a pretrained model, QuPath separated cells into determinants of either tumor or TAS tissue. This expression quantization was then provided as an input for Kaplan–Meier survival models. For both CTLA4 and OX40L, we identified significant differences in relation to biochemical relapse free (BCRF) survival in different tissue types. In tumor cells, high expression of CTLA4 was correlated with a lower overall BCRF survival. However, in tumor-adjacent stroma, high expression of CTLA4 was instead correlated with higher overall BCRF survival. These trends also held true for OX40L expression in both tumor and tumor-adjacent stroma. TMA = tissue microarray; TAS = tumor-adjacent stroma.

Digital Spatial profiling protein expression analysis identified higher expression levels (*p*-value < 0.05 and FDR < 0.2) of two proteins (i.e., CTLA4 and OX40L) and lower expression of CD11c in tumor-adjacent stroma as compared to tumor epithelium (Figure 3). A role for OX40L and CTLA4 and CD11c in cancer immunology and viral infections has been reported [49,50,51,52]. A high expression level of CD11c is associated with better prognosis [52]. The ability to successfully classify patients as either high or low risk for disease progression is of great value for patient management. To investigate this, we used tissue microarrays consisting of 97 total patients with 1547 cores split among different marker staining including CTLA4 (*n* = 514 cores), OX40L (*n* = 522 cores), and CD11c (*n* = 511 cores). We processed the image cores using a cell-detection model that is able to differentiate between stroma and tumor tissue. The model was created using tissue types categorized by a pathologist and was manually verified on 100 additional cores. We then used a Kaplan–Meier survival model to depict the differences between high and low expression of three markers (CTLA4, OX40L, and CD11c) and their effects on the time to biochemical relapse for prostate cancer patients (Figure 4). We found that there was a correlation between high expression of CD11c positivity and increased recurrence time in both tumor and tumor-adjacent stroma (Supplementary Figure S3). For CTLA4, we found a significant variation among the high and low expression thresholds in tumor-adjacent stroma compared to those of tumor tissue (Figure 4A). In tumor-adjacent stroma cells, higher expression was directly correlated with higher biochemical relapse free (BCRF) survival. However, there was an inverse relationship for the expression of CTLA4 in tumor cells, where higher expression was correlated with lower BCRF survival. OX40L followed a similar trend to CTLA4 in which high expression of OX40L was correlated with higher BCRF survival in tumor-adjacent stroma cells, but shorter BCRF survival in tumor cells. Cell type specific expression of CTLA4 and OX40L may predict clinical outcome in PCa patients (Figure 4).

### 3.3. Transcriptome Analysis of PCa Tumor Microenvironment with DSP

The GeoMx Digital Spatial Profiling (DSP) by NanoString uses simultaneous spatial profiling analysis of protein and RNA via ROIs. We used the Cancer Transcriptome Atlas (CTA) panel to profile RNA expression of 1825 genes simultaneous with spatial resolutions from the same 12 regions of interest (ROIs) selected for protein analysis (i.e., TAS, tumor epithelium, CD45+ TAS, CD45+ tumor epithelium) across four PCa patients. The limit of quantification (LOQ) of >2.5 is a threshold recommended by the manufacturer (GeoMx DSP analysis tool, NanoString) to ensure high confidence that the gene is expressed. Of the 1825 genes in the CTA panel, 676 genes had a LOQ > 2.5, and were considered further.

**Figure 5:**
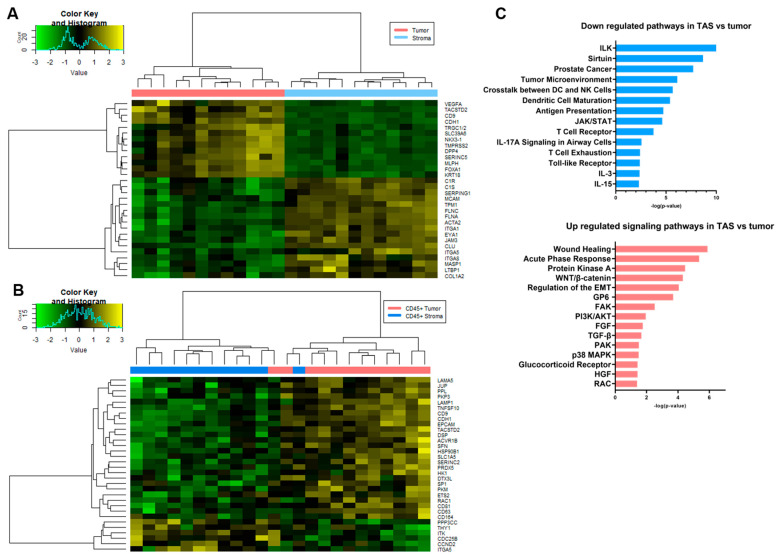
Spatial transcriptomic analysis of tumor-adjacent stroma and tumor. Heatmaps generated using the normalized expression values for genes from the Cancer Transcriptome Atlas, CTA panel for comparisons between (**A**) tumor epithelium and tumor-adjacent stroma and (**B**) CD45+ tumor epithelium and CD45+ tumor-adjacent stroma. The most significant genes (FDR < 0.2 and *p*-value < 0.05) that were differentially expressed among pairs of ROIs are shown. (**C**) Bar diagrams depicting selected significant pathways (*p* < 0.05) associated with downregulated genes and selected significant pathways (*p* < 0.05) associated with upregulated genes in TAS as compared to tumor. Complete pathway analysis can be found in Supplementary Table S6a,b, FDR = false discover rate; ROI = region of interest; TAS = tumor-adjacent stroma.

When TAS (ROI = 12) was compared to tumor epithelium (ROI = 12), 231 genes were differentially expressed at the RNA level (FDR < 0.2 and *p*-value < 0.05) with 162 genes downregulated (e.g., HLA-B, HLA-C, CDH1, MIF, RELA and MDM2) and 69 upregulated (e.g., FZD7, FGFR1, FGF7, FGFR1, TGFBR1, TCF7, FLNA, FLNAC, JAM 3 and CSFR1) in TAS as compared to tumor epithelium (Figure 5A, Supplementary Table S4). Interestingly, comparative pathway analysis using IPA identified several immune responses that were uniquely associated with downregulated genes in TAS as compared to tumor epithelium (−log_10_(*p*-value) > 1.3 or *p*-value < 0.05) including natural killer cell signaling, crosstalk between dendritic cells and natural killer cells, antigen presentation, B cell and T cell signaling, NUR77 signaling in T lymphocytes, CD27 signaling in lymphocytes, IL17A, IL-12, IL-3,IL-13, IL-15, IL-23 signaling, and role of RIG1-like receptors in antiviral innate immunity (Figure 5C, Supplementary Table S6a). Among the pathways (−log_10_(*p*-value) > 1.3 or *p*-value < 0.05) associated with upregulated genes in TAS as compared to tumor epithelium are several signaling pathways that promote tumorigenesis including protein kinase A, WNT/β-catenin, STAT3, FAK, ILK, PI3K/AKT, TGF-B, p38MAPK, HGF, RAC, FGF, and PI3K/AKT (Figure 5C, Supplementary Table S6b). In addition, the regulation of the epithelial–mesenchymal transition (EMT), the role of macrophages, fibroblasts and endothelial cells in rheumatoid arthritis, and the regulation of the EMT by the growth factors pathway were associated with genes with increased expression in TAS (Figure 5C, Supplementary Table S6b). Four genes that were differentially expressed in this panel also overlapped with those found in our previous studies (FGFR1, FLNC, TPM1, FGF7) [53]. 

In a comparison of the differences between CD45+ TAS and CD45+ tumor, the presence of immune cells may result in some of the transcript data collected being diverted to the detection of immune cells, leading to lower representation of rarer transcripts from prostate tissue. As a consequence, the differentially expressed genes and pathways were quite different from the results of the TAS vs. tumor comparison above. The one similarity was that most of the genes differentially expressed at a significant level were downregulated in CD45+ TAS relative to CD45+ tumor (Figure 5B, Supplementary Table S5). Of the 32 genes, 26 (81%) were downregulated in CD45+ TAS including TACSTD2, DSP, PKM, SLC1A5, LAMA5, SFN, and SERINC2. The remaining six genes (e.g., PPP3CC, ITGA5, ITK, CDC25B, CCND2, and THY1) were expressed at higher levels in CD45+ TAS as compared to CD45+ tumor. Pathway analysis of CD45+ TAS and CD45+ tumor epithelium differential expression revealed a number of prostate cancer related pathways including epithelial adherent molecules junction signaling, TEC kinase signaling, and HGF [54,55,56].

### 3.4. The Immune Microenvironment of Leukocyte Infiltrating Tumor-Adjacent Stroma and Tumor Epithelium

**Figure 6:**
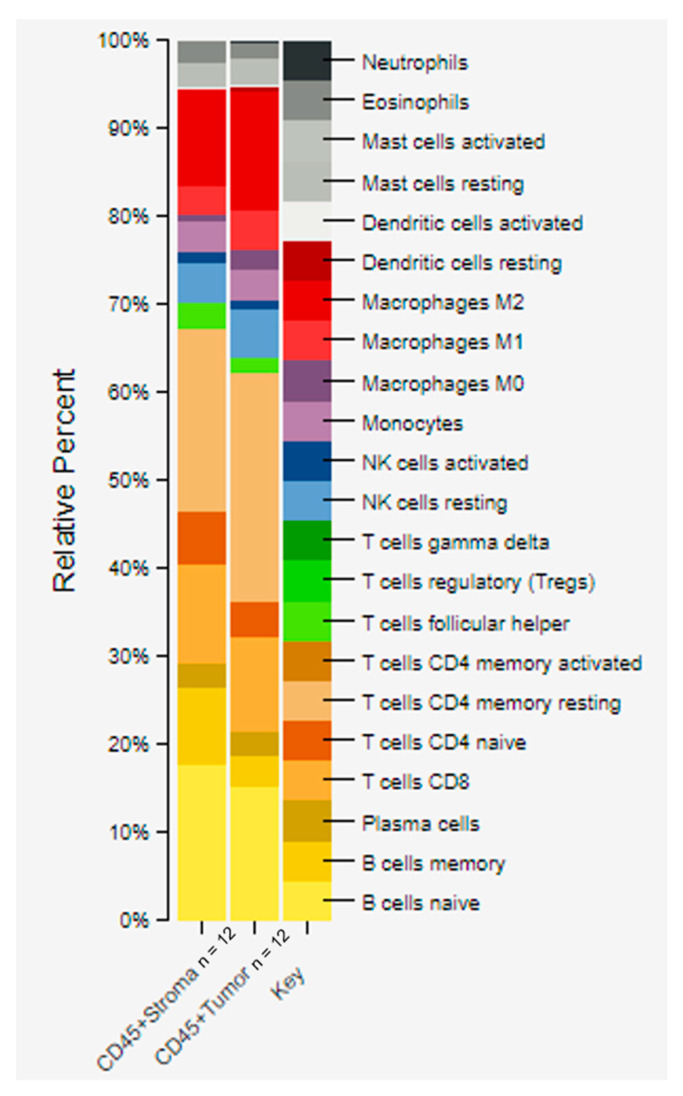
CIBERSORTx analysis of CD45+ stroma and tumor using gene expression data. Quantitative differences were estimated among immune cells from tumor and stroma that contained CD45 positive cells. The stacked bar graphs were generated by providing a normalized gene expression matrix to CIBERSORTx. Higher enrichment in CD45+ TAS compared to CD45+ tumor epithelium was seen in: naïve B cells (18% vs. 15%), memory B cells (9% vs. 3%), and naïve CD4 T cells (6% vs. 4%). In contrast, a higher fraction in CD45+ tumor compared to CD45+ TAS was seen in: memory resting CD4 T cells (26% vs. 21%), M1 macrophages (5% vs. 3%), and M2 macrophages (13% vs. 11%). ROI = region of interest; TAS = tumor-adjacent stroma.

Spatial transcriptomic (i.e., CTA) data was used to estimate the relative fraction of immune cell types across 12 ROIs of CD45+ tumor and 12 ROIs of CD45+ TAS from prostate cancer patients using CIBERSORTx [57,58]. Our data revealed higher average fractions of some immune system cell types in the CD45+ TAS as compared to CD45+ tumor epithelium for both naïve B cells (18% vs. 15%), memory B cells (9% vs. 3%), naïve CD4 T cells (6% vs. 4%) and T follicular helper cells (Tfh) (3% vs. 1%). In contrast, memory resting CD4 T cells (26% vs. 21%), M1 macrophages (5% vs. 3%), and M2 macrophages (13% vs. 11%) were enriched at higher levels in CD45+ tumor epithelium (Figure 6).

## 4. Discussion

Prostate cancer (PCa) remains one of the most diagnosed cancers in men, and yet prognostics remain unreliable, with 25 percent of patients put on active surveillance relapsing within five years [59,60,61,62,63]. Furthermore, prostate cancer patients that experience metastases fail androgen therapy within an average of two years [64,65]. Understanding the biology of the disease and discovering new markers for early detection and therapy are active areas of research [54,66,67]. Furthermore, isolating specific tissue regions may lend itself to discovering alternative therapeutic targets. One potential source of markers and targets is the tumor-adjacent stroma, which has differences in gene expression, genomic aberrations, and molecular features that are potentially exploitable [11,68,69,70,71,72]. For example, we previously identified the diagnostic value of studying tumor-adjacent stroma (TAS) in PCa patients at the RNA level, further promoting the need to explore other molecular differences [7,10].

Here, we utilize digital-spatial profiling (DSP), a robust technique that can quantitatively assess RNA transcript and protein expression levels in formalin-fixed, paraffin embedded samples and provide the ability to sample multiple regions of interest (ROI), to identify variation between tumor and TAS tissue in PCa patients. As CD45 represents an immune cell marker and has been correlated with positive survival in other cancers [73], we also sought to examine the differences that exist in immune cell infiltrated tumor and TAS by applying DSP techniques to CD45+ tissue.

A total of 58 proteins and 1825 genes were quantified using DSP for four PCa patients with three ROIs for each cell type (tumor epithelium, TAS, CD45+ tumor epithelium, and CD45+ TAS). Protein expression analysis identified 24 genes with differential expression in TAS when compared to tumor epithelium at FDR < 0.2 and a *p*-value < 0.05 across the four PCa patients. Higher protein expression levels of surface proteins OX40L and CTLA4 and lower expression of CD11c were detected in tumor adjacent stroma as compared to tumor epithelium. OX40L and CTLA4 are present on multiple antigen-presenting cells, and their higher protein expression has been associated with reduced survival in tumor cells [74]. Conversely, CD11c expression has been positively correlated with higher survival in multiple cancer subtypes [52]. We performed survival analysis using tissue microarray data from multiple PCa patients to analyze the effects of the expression of these proteins on patient outcome in the form of biochemical relapse free survival. Consistent with previous observations [52], we found that CD11c overexpression in both tumor and TAS was positively correlated with increased survival. CTLA4 and OX40L exhibited similar trends to each other, with overexpression in tumor tissue leading to reduced time to relapse, but overexpression in TAS leading to increased time to relapse. The opposing effects that the expression of these two proteins have between the two tissue types for prognostics could prove to be a powerful means of distinguishing aggressive and indolent tumors with one antibody in stained biopsies. The potential for invading immune cells and the stroma to be targets for treatment is of great potential, and the protein expression differences we identified here provide a baseline for future studies. Finally, when examining the proteins differentially expressed between CD45+ TAS and CD45+ tumor epithelium, we identified HER2 as overexpressed in tumor tissue. This protein was found in the same comparison between non-infiltrated TAS and tumor epithelium, consistent with other studies [75]. Furthermore, HER2 overexpression has been associated with breast cancer tumor-infiltrated immune cells via trogocytosis [76]. In fact, recent studies have utilized a scFv-extended IgG fusion to specifically target and degrade the HER2 molecule, thus stifling one of the signaling pathways for tumor cells [77].

Using spatial transcriptomic analysis of the ROIs we identified 162 genes downregulated (e.g., HLA-B, HLA-C, CDH1, MIF, RELA and MDM2) and 69 upregulated (e.g., FZD7, FGFR1, FGF7, FGFR1, TGFBR1, TCF7, FLNA, FLNAC, JAM 3 and CSFR1) in TAS as compared to tumor epithelium. We previously reported down regulation of CDH1 (E- Cadherin) in tumor-adjacent stroma of prostate cancer patients [10]. Deregulation of this gene plays important role in regulating the epithelial–mesenchymal transition that initiates tumor invasion. Low expression levels of CDH1 have also been reported in tumor-adjacent stroma of colon cancer patients [78]. Low expression of HLA class 1 (i.e., HLA-B and HLA-C) indicates impaired priming of cytotoxic CD8+ T cells. In keeping with this, low expression of HLA class I is associated with early prostate cancer recurrence [79]. Abnormal expression of MDM2 in prostate cancer is associated with aggressive behavior [80]. The stromal role of these genes in prostate tumorigenesis is not fully understood. Interestingly, genes of Wnt signaling pathways including FZD7 and TCF7 are upregulated in tumor-adjacent stroma as compared to tumor epithelium. Genes of Wnt pathways are associated with tumor–stroma paracrine interactions [7,12,81].

Spatial transcriptomic analysis of ROIs between CD45+ tissues in both tumor and stroma identified 32 genes that were significantly differentially expressed at FDR< 0.2 and *p*-value < 0.05. CIBERSORTx analysis revealed several key differences among the immune profiles of both CD45+ TAS and CD45+ tumor epithelium. Naive B cells, memory B cells, naïve CD4 T cells and T follicular helper cells (Tfh) cells were relatively enriched in CD45 +TAS whereas CD45+ tumor had higher fractions of resting memory CD4 T cells, M1 macrophages, and M2. Studies of bulk tumor have revealed the cooperation of B and T cells in the tumor may result in induction of anti-tumor immunity [82,83]. This observation is supported by other studies in which the presence of B cells correlated with positive or neutral outcomes in most studies [82]. The increased levels of genes of Tfh cells in CD45+ TAS further supports B cell activation because Tfh cells secrete IL-21, a cytokine that enhances B cell differentiation into anti-tumor antibody secreting B plasma cells. In addition, the presence of Tfh cells is reported to correlate positively with better prognosis in patients with malignant tumors [84].

Conclusion: We have shown differences between tumor and tumor-adjacent stroma (TAS) in prostate cancer, with and without immune cells, using spatial transcriptomic and proteomic techniques. We identified a number of protein and RNA transcript level differences within the tumor microenvironment: STING and CD68 were upregulated in tumors over TAS, suggesting enhanced immune responses targeting specific tissue types. This is an understudied area, and it may be possible that some of the changes such as expression of immune checkpoint inhibitors like OX40L and CTLA4 appear earlier in TAS versus tumor. Thus, it may be beneficial to determine early RNA transcript and protein level changes in TAS as a potential diagnostic marker for progression of prostate cancer. Therapies targeting TAS early in the treatment may also prove to benefit from such analyses.

## Data Availability

Data will be made available on reasonable request.

## Supplementary Materials

The following are available online at https://www.mdpi.com/article/10.3390/cancers14194923/s1, Figure S1: Concordance Plot for Identifying Optimal Normalization Method, Figure S2: Signal to Noise Ratio Plot to Identify Genes of Interest, Table S1: Clinical information for samples used for Digital Spatial Profiling analysis, Table S2: Statistically significantly differentially expressed genes between stroma and tumor ROIs for immune/oncology panel of 58 proteins, Table S3: Statistically significantly differentially expressed genes between CD45+ stroma and CD45+ tumor ROIs for immune/oncology panel of 58 proteins, Table S4: Statistically significantly differentially expressed genes between TAS and tumor ROIs for the Cancer Transcriptome Atlas gene panel, Table S5: Statistically significantly differentially expressed genes between CD45+ stroma and CD45+ tumor ROIs for the Cancer Transcriptome Atlas gene panel, Table S6a: Canonical pathways of 162 downregulated differentially transcribed genes in tumor-adjacent stroma versus tumor epithelium, Table S6b: Canonical pathways of 69 upregulated differentially transcribed genes in tumor-adjacent stroma versus tumor epithelium, File S1: QuPath Trained Model for Identifying Tissue.
