# Supplementary figures and images for: Spatial Profiling of the Prostate Cancer Tumor Microenvironment Reveals Multiple Differences in Gene Expression and Correlation with Recurrence Risk
Source: Cancers (Basel). 2022 Oct 8;14(19):4923. doi: 10.3390/cancers14194923 (PMC9562240; doi:10.3390/cancers14194923)

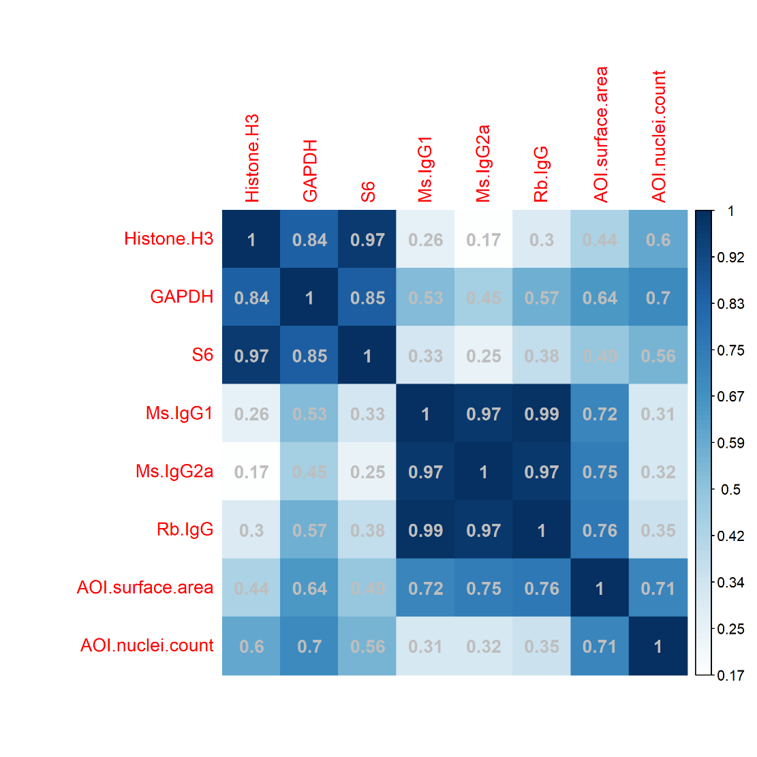

Supplement: Supplementary file 1 [file cancers-14-04923-s001.zip › Supplementary Figure S1. Concordance Plot for Identifying Optimal Normalization Method.png]

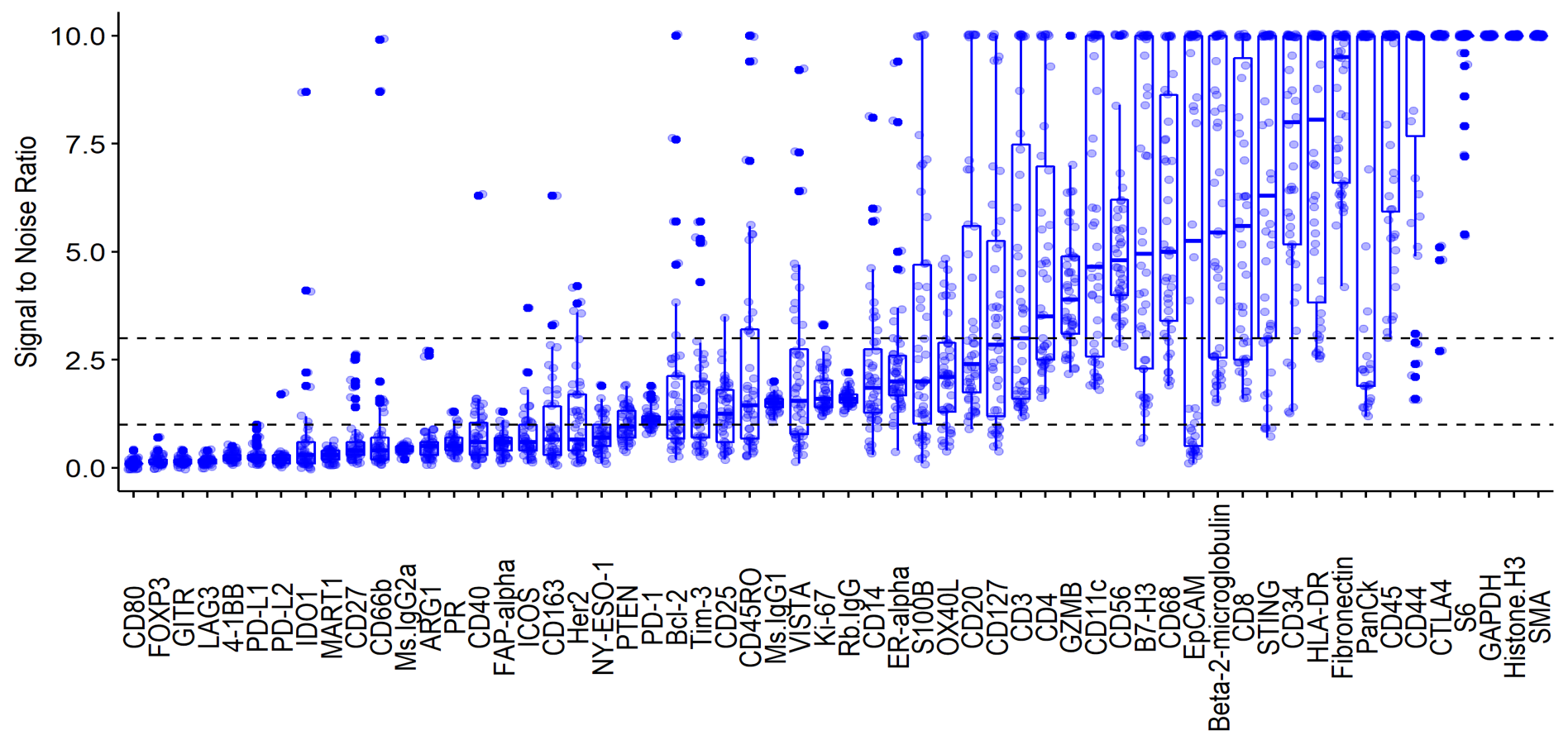

Supplement: Supplementary file 1 [file cancers-14-04923-s001.zip › Supplementary Figure S2. Signal to Noise Ratio Plot to Identify Genes of Interest.png]

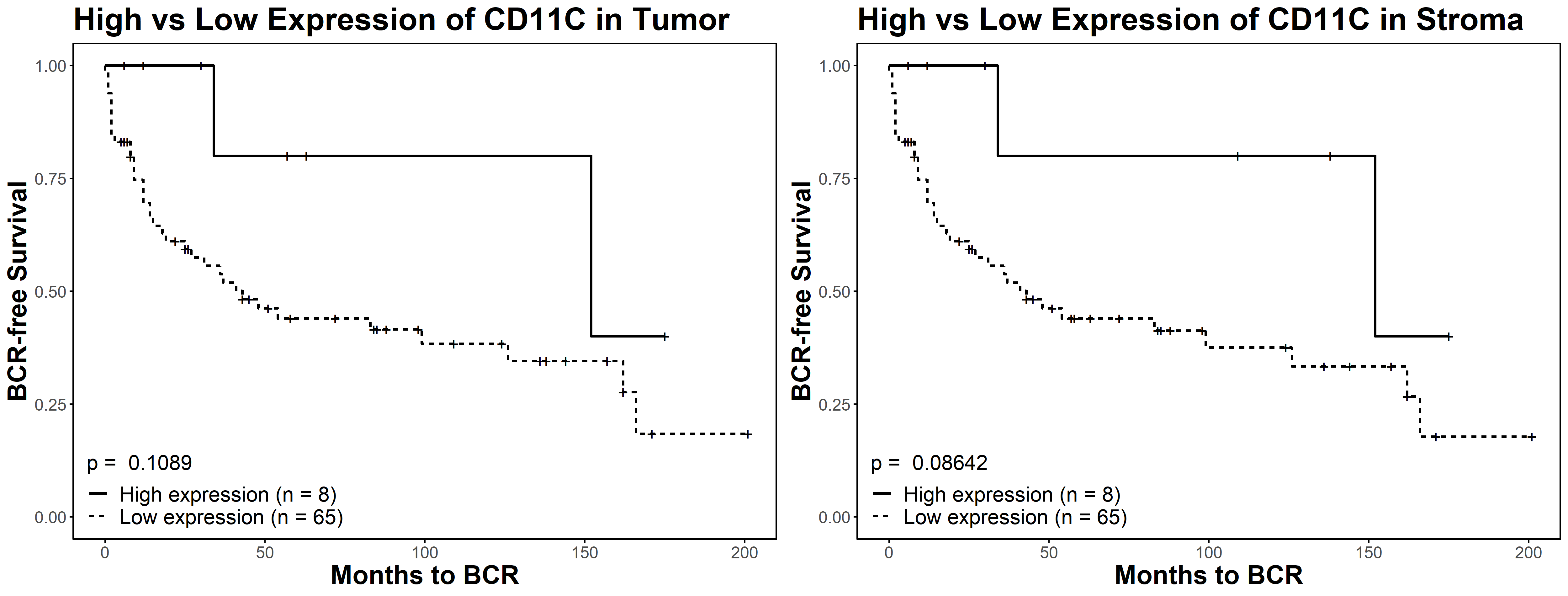

Supplement: Supplementary file 1 [file cancers-14-04923-s001.zip › Supplementary Figure S3.png]
